# Diet Quality Changes by Educational Level among Adults in Spain from 2017 to 2021

**DOI:** 10.3390/nu15040858

**Published:** 2023-02-08

**Authors:** Isabel Romero, Julia Díez, Isabel Del Cura, Manuel Franco, Pedro Gullón, on behalf of the Heart Healthy Hoods Study Group

**Affiliations:** 1 Fundación Instituto de Investigación Sanitaria de Aragón (IIS Aragón), 50009 Zaragoza, Aragón, Spain; 2 Public Health and Epidemiology Research Group, School of Medicine, Universidad de Alcalá, Alcalá de Henares, 28871 Madrid, Spain; 3 Primary Care Research Unit, Madrid Health Service, 28035 Madrid, Spain; 4 Department of Preventive Medicine and Public Health, Universidad Rey Juan Carlos, 28933 Madrid, Spain; 5 Health Services Research on Chronic Patients Network (REDISSEC) & Research Network on Chronicity, Primary Care and Health Promotion (RICAPPS), Institute of Health Carlos III, 28029 Madrid, Spain; 6 Aging Research Center, Department of Neurobiology, Care Sciences and Society, Karolinska Institute and Stockholm University, 17165 Stockholm, Sweden; 7 Department of Epidemiology, Johns Hopkins Bloomberg School of Public Health, Baltimore, MD 21205, USA; 8 Center for Urban Research, RMIT University, Melbourne 3004, Australia

**Keywords:** diet, health inequalities, education, cohort, longitudinal study, adults, Spain

## Abstract

Despite increasing attention on addressing socioeconomic disparities in diet quality, longitudinal studies are scarce. Furthermore, the effects of the COVID-19 pandemic on diet-related outcomes are yet to be fully understood. We examined changes in diet quality by educational level among adults in Madrid, Spain. We used data from recruitment (in 2017) and from 2021. At baseline, our sample included 1358 adults aged 40–75 years who were free of cardiovascular disease and completed a validated diet quality screener. Of them, 931 answered the survey in the follow-up visit in 2021. We used participants' diet quality index scores (range: 18–54; higher scores indicate better diet quality) as the dependent variable. As our independent variable, we assessed participants’ educational levels (low, medium, and high). We fitted a multinomial regression using the categories of educational level as the main predictor, adjusting for age, sex, country of origin, and household composition. During the study period, 78.0% of participants sustained their diet quality, 11.6% improved it, and 10.4% moved away from a healthier dietary pattern. In descriptive analyses, we observed an increase in diet quality among less-educated females. Unadjusted multinomial models showed that a lower educational level predicted both increases and decreases in diet quality over the period. Even though the median diet quality scores did not change significantly, we observed heterogeneous changes over the four years. Variability within diet, with some improving and some worsening, seems to have increased among participants with lower educational levels. Future studies should look at the determinants of change in these population subgroups.

## 1. Introduction

Worldwide, poor diet is the leading risk factor for disease morbidity and mortality and is estimated to result in more than 11 million deaths [1,2]. Indeed, more than 5 million diet-related deaths (45% of total diet-related deaths) occur among adults aged younger than 70 years [2]. These deaths are caused by key behavioral risk factors that are shaped by sociodemographic and epidemiological changes that have occurred in the past decades. 

Unhealthy diets include those high in energy, saturated fat, sodium, refined carbohydrates, or added sugars but low in fruits, vegetables, or whole-grain products [2]. As such, Mediterranean dietary patterns have been shown to promote health and reduce the risk for various diet-related diseases (e.g., type 2 diabetes mellitus) among individuals of all ages [3]. However, countries in the Mediterranean region, such as Malta, Greece, Spain, and Italy, have seen a significant increase in diet-related health problems in recent years, alongside a decline in adherence to Mediterranean dietary patterns [4,5]. In Spain, for instance, this nutrition transition has also made calorically dense foods widely accessible, increasing Spaniards’ consumption of free sugars, saturated fats, and cholesterol.

Dietary behaviors are complex. As a result, it is preferable to examine dietary patterns instead of analyzing individual foods, as they are not consumed in isolation [6]. As such, dietary patterns offer the advantage of describing the typical consumption of foods in a usual diet. Based on this evidence, diet quality scores (DQSs) have been developed to assess the healthfulness of dietary patterns. Thus, DQSs are currently the most common approach to assess adherence to predefined dietary patterns (e.g., a Mediterranean dietary pattern), to examine the association between overall diet quality and health outcomes, or to monitor time trends in dietary scores [6,7]. 

The influences of social factors, such as income, education, gender, or ethnicity, on diet are well documented. Globally, research has shown that these associations follow a clear social gradient in most developed countries: the more vulnerable the individual, the higher the probability of having a poor diet or presenting overweight/obesity [8]. Conversely, the higher the socioeconomic position, the higher the consumption of fruits and vegetables (and the lower the consumption of added fats) [8]. In Spain, compared with low SES, those with higher SES also show an overall healthier diet [5]. Likewise, the prevalence of hypertension, obesity, and diabetes is higher in adults with lower SES [9]. For instance, obesity is three times more common among lower-SES women, while rates among men show little variation [9]. Moreover, educational attainment has been shown to vary the health consciousness related to dietary behaviors [10]. 

Specifically, being older, female, and of a higher socioeconomic position have consistently been suggested to be predictors of improved dietary patterns [8]. In Anglo-Saxon settings, such as Australia or Ireland, research has shown that a higher level of education predicts an improvement in adults’ dietary patterns over time [11,12]. However, this is an issue that remains understudied in Spain.

Furthermore, previous research has shown that some morbidity and mortality rates decrease during economic downturns [13,14]. Indeed, Chen et al. observed how diet quality among US adults improved during the 2008–2009 Great Recession but deteriorated as the economy recovered [14]. A possible mechanism is the substitution of food prepared at home with nutritionally less desirable food away from home during the economic recovery. If this is true, the COVID-19 pandemic may result in even larger changes in diet quality.

Despite all this, few longitudinal studies have examined changes in diet quality over time. Time-trend studies are key to monitoring dietary patterns and to developing public health interventions focusing on those for whom trends are not improving [15]. Thus, understanding changes in dietary quality among adults, and whether and how such changes vary across population subgroups, could uncover dietary inequities and guide interventions to both improve dietary quality and reduce diet-related health outcomes. However, even fewer longitudinal studies have addressed how social inequities are related to changes in dietary behaviors. Thus, this study aimed to examine changes in diet quality among adults in Spain from 2017 to 2021 according to educational level.

## 2. Methods

### 2.1. Study Population

This longitudinal prospective study analyzed data from the Heart Healthy Hoods (HHH) cohort, which was designed to assess the influences of urban determinants on residents’ cardiovascular risk. Detailed descriptions of the HHH study design have been reported elsewhere [16,17]. 

The HHH cohort was developed in collaboration with the Primary Healthcare Service of Madrid, and it recruited men and women aged 40–75 years who lived in the city of Madrid. Recruitment took place from general practice lists, and participants were selected by age, country of origin, and sex-stratified random sampling. Participants visited their primary care health center, where a doctor/nurse collected blood samples and anthropometric measures. Then, they received a call from trained staff, during which they completed a questionnaire on sociodemographic and behavioral variables. All participants provided written informed consent, and the Regional Ethics Committee of Madrid approved the HHH study, which was conducted in accordance with the Declaration of Helsinki.

This study used data collected at recruitment in 2017 and after four years of follow-up in 2021. At the initial recruitment, 1258 participants completed the dietary assessment. By the second visit in 2021, we had lost some participants due to various reasons, including inability to contact (n = 154), refusal to participate (n = 77), relocation (n = 74), or inability to complete the interview (n = 22). Thus, the analytic sample for this study included 931 participants.

### 2.2. Study Variables

As our dependent variable, we measured the change in diet quality between 2017 and 2021. In both waves, we used the Short Diet Quality Screener (sDQS) to assess diet quality. The sDQS, developed by Schröder et al. to estimate overall diet quality in primary-care settings, is a valid measure of diet quality within the Spanish population [18,19]. We chose to use the sDQS to reduce participant burden by decreasing the completion time. The validity of this screener was previously confirmed, and the results suggest that it is a reliable method for assessing diet quality [18,19].

It asks respondents about their usual intake of 18 food items (grouped within three food categories) over the last year. The food items comprised within the first category included major food groups (e.g., bread, vegetables, or fruit). The second category encompassed mainly meat (and other foods of animal origin), and the third category comprised fish, legumes, and nuts. The frequency of food consumption was grouped into three frequency response categories: (1) ‘less than once a day’, ‘once a day’, and ‘more than once a day’ for the eight food items in the first category; (2) ‘less than 4 times a week’, ‘4 to 6 times a week’, and ‘once a day’ for the seven food items in the second category; and (3) ‘less than 2 times a week’, ‘2 to 3 times a week’, and ‘4 or more times a week’ for the three food items in the third food category.

This sDQS produces a diet quality index (DQI) score based on dietary recommendations for the Spanish population. The details of the indices’ development and scoring systems have been described elsewhere [18,19]. Briefly, the daily intake of a portion of foods in the first food group category scored two points, while lower and higher intakes scored one and three points, respectively. Alcohol consumption was scored differently: the consumption of one drink scored three points and lower and higher intakes scored one. In the second group, intake scored two points if reported as 4–6 times per week; while more and less frequent consumption scored one and three points, respectively. High consumption (four or more times per week) of food items of the third food category scored three points; however, intakes of 2–3 times and less than twice a week scored two and one points, respectively. Then, all food item scores were added up. We divided the final score of the sDQS (which ranged from 18 to 54) into two categories: inadequate or semi-adequate diet quality (DQI scores of 18–42) vs. adequate diet quality (DQI scores of 43–54).

Our main independent variable was the participants’ socioeconomic status, which we measured using their educational level as a proxy [20]. Following the International Standard Classification of Education (ISCED), we collapsed this variable into three categories (low, medium, and high levels of education) [21]. In addition, participants reported their sociodemographic information, including age (as a continuous variable), sex (categorized as male/female), country of birth (Spain/outside of Spain), and the number of people living in their household.

### 2.3. Statistical Analyses

We conducted our analyses between November and December 2022 using STATA 15 (StataCorp, CollegeStation, TX, USA) and R software. First, we carried out descriptive analyses to provide an overview of participants’ characteristics concerning their diet quality in 2017 and 2021. Then, we used multinomial regression models to assess the change in diet quality (increasing or decreasing between 2017 and 2021) with their educational level (using participants with a high educational level as the reference group). Initially, we fitted the crude models, which we then adjusted with the following potentially confounding variables: age (as a continuous variable), sex (categorized as male/female), country of birth (Spain/outside of Spain), and the number of people living in each household. Since there is evidence of sex-specific determinants of dietary behaviors, we carried out regression analyses first on all participants and then stratified by sex [22].

## 3. Results

### 3.1. Participants’ Characteristics 

Table 1 shows the descriptive statistics. The sample for this study included 931 adults, of which 56.5% were women, 47.8% were between the ages of 40 and 55, 83.8% were born in Spain, 38.8% had a higher education level, and 12.1% lived alone.

### 3.2. Crude Trends in Diet Quality

**Figure 1:**
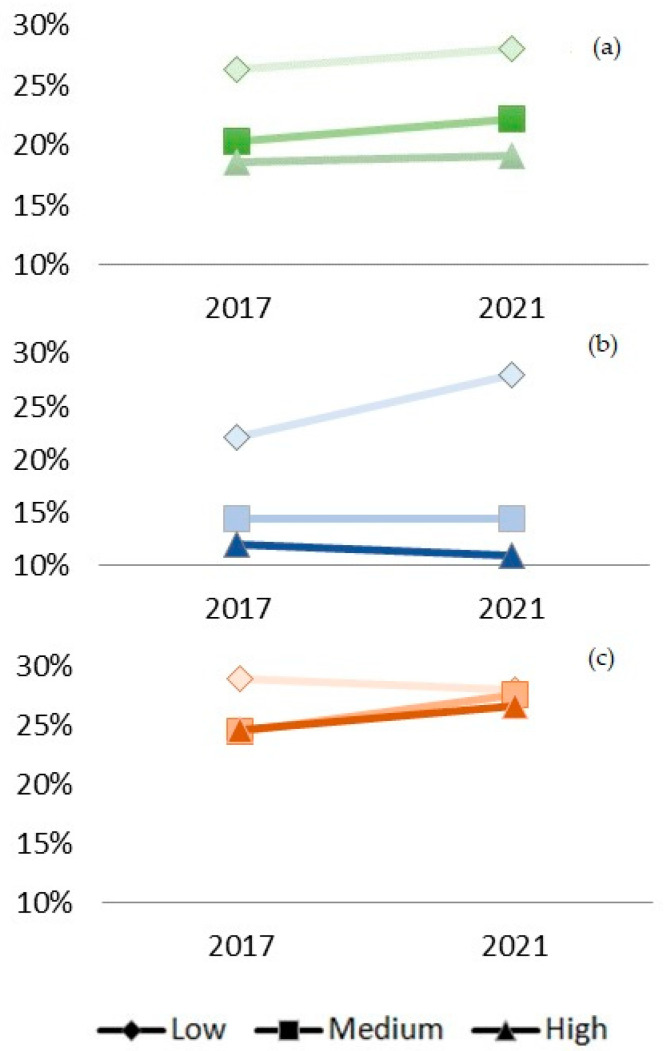
Crude prevalence of an adequate diet (DQI score > 42) by educational level among adults in Madrid from 2017 to 2021: (**a**) overall; (**b**) for women; (**c**) for men.

From 2017 to 2021, 78.0% of participants sustained their diet quality, 11.6% improved it, and 10.4% moved away from a more adequate dietary pattern. Figure 1 illustrates the descriptive changes in the diet quality categories by educational level within the study period, as described in the text. Overall, the prevalence of an adequate diet slightly increased among participants with low and medium educational levels. The prevalence of an adequate diet increased among women with a low educational level. Among men, diet quality increased for those with a medium or high educational level, but it slightly decreased for those with a low educational level. 

### 3.3. Predictors of Change in Diet Quality 

Table 2 shows the association between educational level and improvement in diet quality from 2017 to 2021. In the unadjusted models, participants with a low educational level had 85% increased odds of improving their diet quality (95% confidence interval (CI) = 1.06–3.11) compared to those with a higher educational level. After adjusting for potential confounders, the direction of the association remained, but it lost its statistical significance (odds ratio (OR) = 1.70; 95% CI = 0.96–3.02). The results were consistent across males and females, with both groups showing increased odds of improving their diet quality when compared to those with a higher educational level. However, the sex-stratified results did not reach statistical significance in the unadjusted or the adjusted multinomial regression models.

Table 3 shows the association between participants’ educational levels and the worsening in their diet quality between 2017 and 2021. Participants with a low educational level had increased odds of decreasing their diet quality in both the unadjusted and adjusted models. However, these associations did not reach statistical significance for the overall sample. We observed a similar association among males. However, this was not statistically significant. We found stronger associations among females, where a low educational level predicted a decrease in diet quality in the unadjusted model (OR = 2.15; 95% CI = 1.04, 4.45). Once adjusted for potential confounders, the direction of the association remained but lost its statistical significance (OR = 1.45; 95% CI = 0.65–3.21).

## 4. Discussion

This study evaluated time trends in diet quality in a cohort of adults in Spain and examined whether educational level predicted these changes over the four years of follow-up. Overall, we found that most participants maintained the same diet quality over the four years of follow-up. This is consistent with previous research [15,23] and with the plateauing, but not reversing, of obesity rates among adults [24]. To our knowledge, this is one of the few studies that has examined the influence of educational level on diet quality over time in Spain.

In our descriptive analyses, we observed an inverse pattern between participants’ educational level and their diet quality. Indeed, the prevalence of an adequate diet was higher among participants with a lower educational level. Specifically, we observed an increase in diet quality among less-educated females. Among males, diet quality increased for those with a medium or high educational level, but it slightly decreased for those with a low educational level. In the unadjusted multinomial models, we observed that a lower educational level predicted both increases and decreases in diet quality over the period. This means that there are heterogeneous changes associated with this period; variability in diet (some improving and some worsening) could have increased in participants with lower educational levels. 

Previous research, conducted mostly in the US, has shown that adults with low levels of educational attainment have poorer diets [25]. Furthermore, as observed by Rehm et al., the gaps in diet quality by income and education levels have widened over the past decade [25]. However, the evidence for this relationship in other settings is mixed. In Australia, Baldwind et al. did not observe an association between higher socioeconomic status and greater increases in diet quality [15]. In Spain, for example, a previous study observed no differences in adherence to a healthier dietary pattern by educational level [26]. 

Multiple factors, such as cultural traditions, targeted advertising, and food availability and affordability, may contribute to shaping diet quality among adults, regardless of their educational level or socioeconomic status. As such, we speculate that lower prices of healthy items (e.g., fruits and vegetables) in Spain, compared to the lower affordability of the same items in the US, might play a role in this context-specific association.

Our study sample included only middle-aged and older adults, and a more age-diverse population could show different patterns, as there are cohort effects in dietary patterns. Indeed, previous studies have shown mixed results in relation to age and diet quality. While some have reported increased diet quality with age [27,28,29], others have observed a decrease [30].

Moreover, previous research has stressed how educational attainment can explain dietary inequities beyond financial constraints [31]. Indeed, educational inequalities in dietary behaviors could also be partially explained by cultural capital. Educational attainment can provide the tools to both access and understand food-related knowledge. Furthermore, the social diffusion theory suggests that people from higher educational levels tend to take up innovations sooner than less-educated individuals [32]. This might also occur when more-educated groups try the most novel yet unhealthier food products. As such, we argue that it may be the consumption of unhealthy products and not the decrease in the adherence to a Mediterranean dietary pattern that might be driving dietary inequalities in a Mediterranean context such as Spain.

Furthermore, previous research has shown that increases in diet quality scores among those with an initial poorer diet quality are associated with a lower mortality risk [33]. 

This longitudinal study contributes to our understanding of the predictors of dietary patterns in adults, which seem to be highly context-dependent. To date, most research examining the influence of educational level on changes in diet among adults has relied on studies conducted in Anglo-Saxon settings. For example, Thorpe et al. observed that higher education predicted favorable changes in dietary patterns among Australian adults [23]. These results were in line with other studies conducted in the UK [34]. However, most studies have assessed diet cross-sectionally, and more longitudinal studies exploring changes in dietary patterns are warranted. 

Our results are particularly relevant in the context of the COVID-19 pandemic, as the impacts of the lockdown on diet-related outcomes are still uncertain. In this sense, a recent systematic review examined changes in dietary behaviors (snacking, fast food, and ordered food) during the lockdown period [35]. Although the results showed a significant decrease in fast-food consumption, this review did not assess whether these behaviors and changes differed according to participants’ socioeconomic status. In the UK, a recent population-based cohort study estimated changes in fruit and vegetable consumption and observed a decrease during the pandemic, which was greatest in women and the most socioeconomically disadvantaged participants. 

Interestingly, other studies were in line with our study. For example, Canadian researchers found that diet quality slightly improved, especially among participants with lower education, during the COVID-19 pandemic [36]. In Mexico, researchers found an improvement in diet quality during confinement [37]. In Spain, a previous cross-sectional study observed that adherence to the Mediterranean diet improved during this period [38]. These unexpected results may be partially explained by the greater access to homemade foods during the lockdown. The pandemic gave opportunities, especially among less-educated individuals, to allocate more time to food preparation. Moreover, the nutritional quality of food consumed away from home tends to be lower compared with food prepared at home (e.g., fewer servings of whole grains or vegetables). More frequent cooking at home is associated with better diet quality among both lower- and higher-SES adults [39]. These results emphasize the significance of monitoring diet quality and disparities during times of crisis, as events such as the COVID-19 pandemic can significantly alter health behaviors, including diet. While individual changes may seem small, the overall impact on population health can be significant, a phenomenon known as the prevention paradox [40]. 

We specifically observed that the association of educational level with diet quality was different in men and women. Previous research has also observed that diet-related gender roles (e.g., food-preparation abilities) are only positive predictors of diet quality changes among older women [10]. Another possible reason for these gender-specific differences may be the scoring system of the diet quality screener. For instance, moderate alcohol consumption contributed positively to the total score. Thus, we speculate that men in our cohort were “protected” by their female partners who facilitated their adequate food intake while procuring and preparing meals. 

This study highlights the importance of developing interventions to improve diet quality in vulnerable population subgroups, even though the reasons and mechanisms behind changes in diet quality are not fully understood. These interventions should acknowledge and address the existing socioeconomic barriers to changing dietary behaviors and focus on those most likely to benefit those with lower SES. Our results should be included into the body of literature evaluating diet quality and social inequities in diet quality among different countries. Additionally, the findings of this study emphasize the potential for middle-aged participants with low diet quality scores to reduce their cardiovascular risk by improving their diets. The short-term changes observed in this analysis provide a more realistic and practical evaluation of the dynamics of diet quality. Further research is required to investigate the potential underlying social factors that influence diet quality as well as to identify and address any obstacles that may prevent individuals at risk of poor diet quality from consuming healthier foods.

### Strengths and Limitations

This study has several strengths. While much of the literature reports on cross-sectional determinants of diet quality, we had the opportunity to examine educational attainment as a determinant of diet quality over four years of follow-up. Moreover, changes in diet during the COVID-19 pandemic have mostly been analyzed using repeated cross-sectional analyses, which are not able to observe the individual changes in diet quality that we were able to measure. Moreover, we included a prospective population-based design and repeatedly validated dietary data.

Our study also presents limitations. First, as in all studies, the diet quality data were self-reported. Thus, participants might have under- or overestimated their dietary behaviors. If individuals from lower educational levels felt ashamed or embarrassed about reporting that they had poor dietary habits, even if they were aware that they should be eating healthier but were unable to do so, this could have led to the inaccurate reporting of dietary quality and skewed our results towards the null. 

Moreover, the diet quality screener comprised a restricted number of food items. Additionally, it is possible that different predictors of change exist for the separate components of diet quality. However, previous research has emphasized the usefulness of short dietary assessments in identifying individuals' cardiovascular risk [12]. Further, these short screeners are very efficient in time-limited settings such as primary healthcare. 

Second, there is the potential for confounding factors that might have changed over time and that may also be related to both dietary behaviors and educational level. For example, changes such as unemployment or divorce may be associated with both diet quality and participants’ educational level. Moreover, we did not assess changes in physical activity levels. Third, the follow-up period was relatively short, and this might have influenced the changes observed in diet quality. However, this time period allowed us to assess the effect of the COVID-19 pandemic. An additional limitation to this research is the grouping of heterogenous age groups in order to maintain statistical power, despite the expected variations within these groups (e.g., younger adults vs. retired adults). 

Lastly, we did not assess whether changes in diet quality were associated with consequent changes in body weight. This association has been observed to be stronger among younger age groups [41]. 

## 5. Conclusions

In conclusion, we found that overall diet quality did not change between 2017 and 2021 in our cohort of adults living in Madrid, Spain. Our results showed that having a lower educational level predicted both an increase and a decrease in diet quality, particularly among women. Our findings underline the importance of monitoring socioeconomic and gender disparities in diet, especially in situations of social upheaval, such as the COVID-19 pandemic, which can alter dietary behaviors. Further research should investigate the underlying mechanisms behind these changes in dietary patterns during the pandemic and whether these effects are reversible over time. Future studies examining socioeconomic factors and time trends in relation to diet quality, as well as their interactions, are needed. 

## Figures and Tables

**Table 1:** Sample characteristics by diet quality in Madrid from 2017 to 2021 (n = 931).

Characteristics, % (N)	Semi-Adequate Diet Quality (DQI Scores 18–42)	Adequate Diet Quality (DQI Scores > 42)
Sex		
Men	84.7 (343)	15.3 (62)
Women	72.6 (382)	27.4 (144)
Age group		
40–55	81.3 (395)	18.7 (91)
56–65	72.0 (183)	28.0 (71)
66–75	77.5 (147)	22.5 (44)
Country of birth		
Spain	78.2 (610)	21.8 (170)
Outside of Spain	76.2 (115)	23.8 (36)
Educational level		
Low	72.0 (131)	28.0 (51)
Medium	77.9 (299)	22.1 (85)
High	80.9 (292)	19.1 (69)
Household composition		
One person	85.8 (97)	14.2 (16)
Two people	73.9 (235)	26.1 (83)
Three people	81.3 (191)	18.7 (44)
>Three people	80.4 (213)	19.6 (52)

**Table 2:** Association between educational level and improvement in diet quality among adults in Madrid from 2017 to 2021.

Educational Level	OR *	95% CI *	OR	95% CI
	Unadjusted		Adjusted *	
Overall (N = 931)				
High	1 (ref)		1 (ref)	
Medium	1.21	0.78–2.00	1.19	0.73–1.92
Low	1.85	1.06–3.11	1.70	0.96–3.02
Men (N = 405)				
High Medium Low	1 (ref)		1 (ref)	
	1.00	0.47–2.17	1.01	0.46–2.21
	2.08	0.90–4.83	1.90	0.78–4.61
Women (N = 526)				
High	1 (ref)		1 (ref)	
Medium	1.38	0.76–2.53	1.31	0.69–2.45
Low	1.62	0.80–3.27	1.64	0.77–3.52

* OR: odds ratio; CI: confidence interval; adjusted by age, sex (for the overall models), country of birth, and household composition.

**Table 3:** Association between educational level and worsening in diet quality among adults in Madrid from 2017 to 2021.

Educational Level	OR *	95% CI *	OR	95% CI
	Unadjusted		Adjusted *	
Overall (N = 931)				
High	1 (ref)		1 (ref)	
Medium	1.12	0.68–1.83	0.91	0.54–1.56
Low	1.72	0.99–3.01	1.23	0.68–2.22
Men (N = 405)				
High Medium Low	1 (ref)		1 (ref)	
	0.89	0.42–1.87	0.72	0.33–1.56
	1.17	0.47–2.99	0.80	0.30–2.16
Women (N = 526)				
High	1 (ref)		1 (ref)	
Medium	1.34	0.96–2.62	1.03	0.51–2.09
Low	2.15	1.04–4.45	1.45	0.65–3.21

* OR: odds ratio; CI: confidence interval; adjusted by age, sex (for the overall models), country of birth, and household composition.

## Data Availability

Data are available upon request.

## Supplementary Materials

The following supporting information can be downloaded at https://www.mdpi.com/article/10.3390/nu15040858/s1, the complete list of collaborators and members of the Heart Healthy Hoods cohort study group (surname is reported in bold).
