# Supplementary material for: Diet Quality Changes by Educational Level among Adults in Spain from 2017 to 2021
Source: Nutrients. 2023 Feb 8;15(4):858. doi: 10.3390/nu15040858 (PMC9961002; doi:10.3390/nu15040858)
Supplement: Supplementary file 1 [file nutrients-15-00858-s001.zip › nutrients-2116675-supplementary.pdf]

Complete list of collaborators, members of the Heart Healthy Hoods study group

(surname is reported in bold)

Maria **Urtasun**, Angélica **Bonilla**, Esperanza **Escortell**-**Mayor**, Juan Carlos **Gil-Moreno**, Luis **Sánchez-Perruca**, Antonio **Díaz-Holgado**, Elena **Polentinos-Castro**, Alba **Cebrecos**, Xisca **Sureda**, Usama **Bilal**, Luis **Cereijo**

Clinical investigators from the Primary Care Health Centers (PCHC):

PCHC Los Alpes: Gustavo Mora Navarro, Maria Luisa Ayuso Algora, Lourdes Botanes Peñafiel, Mª Begoña Brusint Olivares, Raquel Cabral Rodriguez, Ninfa Castillo Biscari, Miriam Castro Benito, Mª Ascensión Delgado Lopez, Ana Belen Garcia Da Silva, Maria Victoria Garcia Espinosa, Celia Garcia Garcia, Ana Maria Garcia Ortega, Mª Jose Guereña Tomas, Blanca Jerez Basurco, Mª Dolores Martin Alvarez, Ana Isabel Moreno Gomez, Catalina Ogaya Chica, Belen Ortiz Goncalves, Sara Ascensión Perez Medina, Isabel Prieto Checa, Margarita Puerto Rodriguez, Mauricio Sainz-Maza Aparicio, Oscar Sanchez Lopez, Ignacio Sevilla Machuca, Natalia Vara Reviejo, Mª Dolores Velazquez Martin, Maria Belen Vicente Mata, Mª Pilar Vich Perez. **PCHC Ángela Uriarte:** Jose Antonio Pere Pedrol, Maria Oliva Fernandez Diez, Maria Dolores Gonzalez Biosca, Lucia Iglesias Lopez, Maria Luisa Iglesias Rodriguez, Maria Teresa Mansilla Martin, Rafael Prado Sanchez, Marta Rosario Reina Sánchez, Maria Pilar Rubio Diez. **PCHC Artilleros:** Maria del Carmen Martin de Vidales Hernandez Maria Begoña Ayuso De la Torre, Ines Casado Mora, Eva Maria Donaire Jimenez, Maria Guadalupe Garcia Caro, Maria Carmen Gonzalez Benito, Laura Hernan-Perez Torralba, Riansares Lopez Palomar, , Ana Valvanera Villegasa Arroyo. **PCHC Barajas:** Irma María Ramos Gutierrez, Margarita Camarero Shelly, Sofia Causin Serrano, Julian Diaz Sanchez, Maria Dominguez Paniagua, Maria Norma Fernandez Alonso, Guadalupe Garcia Martin, Rosa Gomez Del Forcallo, Inmaculada Gonzalez García, Jorge Iglesias Vazquez, Francisco J. Martin Martin, Mª Cristina Montero García, Anita Panizo Bayo, Marina Marta Prado Calvete, German Reviriego Jaen, Francisca Rodriguez Lorite, Josefa Mª San Vicente Rodriguez, Mª Pilar Serrano Simarro. **PCHC Caramuel:** Juan Carlos Gamboa Puñal, José Bermejo Vicedo, Ana María Blasco Martín, Beatriz Bueno Rodriguez, Silvia Maria Carmena Diaz, Carlos Comín Mañogil, Isabel Del Valle De Joz, Concepción García Valverde, Sara Gil Bolaño, Maria Teresa Nieto Pomares, Virginia Perez-Oviedo García. **PCHC Cerro Almodóvar:** Maria Mercedes Irazazabal Velasco Eva Barrios Martos, Ana Belen Blazquez Blazquez, Alina Dumbraveanu , , Mercedes Martinez Orozco, Maria Rosario Perez Manzano, Sara Ruiz Ordoñez, Ana Isabel Salamanca Sanz, Maria Elena Vaquero Fernández, Francisca Vico Martinez. **PCHC Ciudad Jardín:** Carmen Lopez Rodriguez, Ana Maria Arias-Salgado Robsy, Camila Elena Arranz Villalva, Mª Jose Calatrava Trigueros, Maria Angeles Cava Rosado, Inmaculada Cuevas Lopez, Yolanda Entrena García, Maria Elvira Herrero Sancho, M.D.L. Angeles Jaime Siso, Mª del Mª Lourdes Martinez Gonzalez, Gregoria Rodriguez Castro. **PCHC Daroca:** Antonio Cabrera Majada, Mercedes Ines Becerra Gonzalez, Carmen De la Riva Salmeron, Ivan De los Mozos Hernando, Nuria Jimenez Olivas, Beatriz Elena Lopez de Mingo, Tatiana Lozano Fernandez, Olga Muñoz Ayuso, Cristina Reyero Montes, Maria Encinar Santano Amores, Mª Luisa Vacas Roldan, Iziar Vazquez Carrión. **PCHC Dr. Castroviejo:** Mercedes Parrilla Laso, Alvaro Carrillo Fernandez, Mª Angeles Diaz Entresotos, María Fernandez Castellanos, Esther Frias Diaz, Ana García Martinez, Isabel López Sanchez, , Maria Paz Perez Unanua, Margarita Ruiz Pacheco. **PCHC Eloy Gonzalo:** Bonifacio de Esteban Marfil, Enrique Roberto Duran Garrido, Sonia Luna Ramirez. **PCHC Entrevias:** Belen De Llama Arauz, Margarita Alvarez Domingo, Maria De Miguel Vicenti, Maria del Mar Duque Herraiz, Angelica Encinas Gonzalez, Laura Fernández Díez, Iciar

Fernandez de Alarcon Medina, Ana Isabel Gonzalez Cebreros, Maria Rocio Lopez Recio, Daniela Milcheva Ivanova, Inmaculada Concepción Morales Murillo, Maria del Carmen Villar Herguedas, Haijun Zhou Chen. **PCHC Espronceda:** Roberto Cabrera Velez, Juliana Alzate Gallego, M. José Castillo Lizárraga, Yolanda Diez Garcia, Maria Carmen Belen Garcia Fortea, Isabel Hernandez Navarro, Aurora Montserrat Martin Diaz, Paloma Pujol Bengoechea, Isabel Saenz Del Castillo Vicente, Lorena Solorzano De Pablo. **PCHC Federica Montseny:** Maria Sonsoles Moreno Muñoz, Sara Ares Blanco, Raquel Blanco Corral, Isabel Díaz Feito, Maria Jesus Fidalgo Baz, Maria Yolanda Hernando Sanz, Silvia Morcillo De la Cuadra, , Estrella Moya Tordesillas, Monica Sánchez Gonzalez, Mª Isabel Vaquero Fuentes. **PCHC Fuencarral:** Alejandra Montero Costa, Mercedes Adrian Sanz, Miguel Angel Alvaro Sanchez, Isabel Caceres Tome, Maria Angustias Calle Rivas, Maria Dolores Cano Perez, Jesica Corona Barrio, Silvia Jimenez Maillo, Maria Pilar Lopez Morandeira, Erika Lutz García, Pilar Martin Aranda, Maria del Carmen Mateo Pascual, Alvaro Ortega Pascual, Lourdes Poza Fresnillo, Estrella Robles Fernandez, M. Pilar Rubio Sanz, Maria Rosario Sanz Merino. **PCHC García Noblejas:** Almudena Uranga Gomez, Mª Aranzazu Alonso Leonardo, Esmeralda Alonso Sandoica, Mª Eugenia Calonge Garcia, Mª Teresa Cuenca Blanco, Maria de la O Escribano Paez, Concepción Falcón Algarra, Teresa Galan Gutierrez, Adelaida Iglesias Docampo, Maria Victoria Izquierdo Delgado, Silvia Medrano Sanchez, Cristina Olmos Sancho, Juana Pascual Conejo, Pilar Perez Elías, Maria Angeles Rodriguez Sierra, Rosario Ruiz Giardin, Maria Cristina Santos Alvarez, Julio Isidro Turrientes Garcia Rojo, **PCHC General Ricardos:** Isabel Alba Llacer, Francisco Ramón Abellan Lopez, , Olga Alvarez Montes, Marta Bosom Velasco, Carlos Casado Alvaro, Pilar Chaves Sanchez, Mari Carmen De las Heras Gomez, Sara Teodosia Fernandez Redondo, Maria Celeste Garcia Galeano, Jorge ignacio Gomez Ciriano, Alicia Hurtado Lambas, Jacinto Macias Rodriguez, Raquel Mateo Fernandez, Gema Moreno Fernández, Maria Luisa Riestra Martinez, Yolanda Rodriguez Garcia, Rosa Maria Rodriguez Moreno, Maria Eloisa Rogero Blanco, Cesar Sanchez Arce, Isaias Utiel Bermejo. **PCHC Guayaba:** M. Concepcion Vargas-Machuca Cabañero, Paloma Aguilera Reija, Cecilia Aragón Marente, Maria Luisa Carnicero Martinez, Carmen Coello Alarcon, Santiago Carlos De la Misericordia Garcia, Belén Garcia Muñoz, Paloma Garrido Calleja, Sonia Gil Rodriguez, María Jesús Heras Alonso, Francisco Javier Martinez Suberviola, Andrea Nieto Jurado, Montserrat Ramos Ruano, Mª Pilar Rodriguez Perulero,. **PCHC Ibiza:** Jorge Olmedo Galindo, Virginia Antolin Diaz, Stephanie Cantillo Torres, Maria Jose Concejo Carranza, Justo Fernandez García, Antonio García Romero, Adela Gavela Perez, Susana Guijarro Valverde, Marta Guil Torres, César Lesmes Lora, Elizabeth Rodriguez Cerón, Maria Isabel Rodriguez de Guzman Barbero, Margarita Isabel Serrano Heras, Paloma Tutor Mansino. **PCHC Lagasca:** Maria de los Angeles Ruiz Morales, Avelino Alvarez Pravia, María Elena González Lucas, Jesus Gorosabel Rebolleda, María Estrella Mediavilla Hernando, Mercedes Palmeiro Castellana, Noelia Polo Fernandez, Juan Ignacio San Vicente Domingo. **PCHC Las Cortes:** M. Jose Gomara Martinez, Marcos López Carrasco. **PCHC Lavapies:** Rosa Maria Bajo Viña, M. Carmen Alvarez Orviz, Carmelo Álvaro Almedros, , Maria Esther Baz Gervas, Araceli Blazquez De la Calle, Delia Cava Julián, Consuelo Civera Aguado, María Luisa Fernandez Monsalve, Teresa Fontecha Gutierrez, Francisco Javier Garcia Oliva, Natalia Gil Garrido, Jesus Herrero Hernandez, Antonio Jimenez Caño, María Jesús López Rodríguez, Cristina Magaz Leones, Manuel Martín Bernal, Obdulia Paloma Martinez Carrion, Esther Martinez Lago, Obdulia Paloma Martínez Carrión, Celia Pecharromán Sacristán, M Teresa Pinedo Moraleda, M Carmen Sánchez Ramos, Emilio Serna Martinez, Carolina Tessainer Cabellos-Nocq, Maria Belen Torres Labandeira. **PCHC Los Yébenes:** Alejandro Rabanal Basalo, Ana Maria Abad Esteban, Pilar Alonso Alonso, Maria Sofia Bellido De Vega, Rosa Eva Callejo Del Pozo, Encarnacion Campillo Marcos, M. Carmen Carballido Garcia, Raquel Carretero Ramos, Yolanda Garcia Villareal, Beatriz Garcia-Serrano Jimenez, Carmen Aurora Garcia-Tenorio Damasceno, M. Teresa Gijon Seco, Alicia

Dolores Horcajada Guijarro, Maria Paz León Fernandez, Maria Soledad Lopez Lozano, Rebeca Mielgo Salvador, Maria Mercedes Navarro Pablos, Maria Josefa Perez Bonilla, , Maria Jose Roldan Parra, M. Angeles Rollan Hernandez, Milagros Saguillo Antolin, Ana Maria Sanchez Martin, Belén Sánchez Moreno, Milagros Santiago Jimenez. **PCHC Mar Báltico:** Rafael Alonso Roca, Mª Nieves Caparros Ezpeleta, Mª Carmen Castillo Lopez, Tirso Galiano Arroyo, Maria Jesus Huerga Gonzalez, Maria Raquel Lopez Del Cid, Mª Carmen Reyes Madridejos, Mª Teresa Sanchez-Villares Rodriguez, Esperanza Villar Coloma. **PCHC Monovar:** Jose Ignacio Vicente Diez. Elsa Maria Burgos Costalago, Alberto Curiel Blanco, Luis Miguel Gomez Garzon, Maria Gomez Martin, Maria Teresa Henandez Holgado, Raquel Juez Pimienta, Isabel Marchante Fernández, Esther Vaquero Lucas, **PCHC Pavones:** Mercedes Ibañez Brillas, Ana María Alonso Blanco, Sonia Atienza Perez, Sonia Canora Gonzalez, Agustina Criado Alcazar, Pilar Cruz Robles, Miriam Garrote Garcia, Aida Virginia Gómez Díaz, Luis Miguel Gonzalez Perez, Maria Inmaculada Hernanez Beltran, Maria Huertas Uhagon, , Juan Ramon Iglesias Quintana, Purificación Magan Tapia, Maria de la Peña Martin Francisco, David Villamañan Lobo. **PCHC San Andrés:** Rosa Maria Gonzalez San Segundo, Luis Alaman Barbero, Enrique Blaya Lopez, M. Luisa Enriquez Lopez, M. Jesus Ferrer Signes, Julia Herranz Hernando, Blanca Jimeno Aguado, Pilar Lopez Rogel, Jose Maria Molero Garcia, David Molina Gomez, Sara Isabel Moreno Puertas, Patricia Navarro Gil, Pedro Otones Reyes, Alejandra Blanca San Antonio Gil. **PCHC San Fermín:** Cristina de Alba Romero, Juan Manuel Cabello Barrios, Mª Victoria Cierva Sardina, Maria Ciprian Tejero, Esther Fernandez Castro, Lourdes Gonzalez Alejandre, Maria Luisa Gonzalez Alonso, Ricardo Gonzalez Tejada, Manuel Hernández Larrea, Olga Luisa Martinez-Manglano Parra, Maria del Rosario Molina Cabrerizo, Purificación Nistal Justel, Rosa Ortega Reinoso, Mercedes E. Ortiz del Amo, Maria Poza Anton, Maria Cristina Rincon Parra, Rosa Rodriguez Fernandez, Susana Rodriguez Fernandez, Pilar Rodríguez Prieto, M. Pilar Sainz Camuñas. **PCHC Torito:** Angela Castro Cordoba, Juan Angel Cique Herrainz, Maria Rosario Duran Tejeda, María Rosa García Redondo, Yolanda Hidalgo Calleja, Juan Manuel Morales Rendon, Maria Mercedes Redondo Sanchez. **PCHC Valdebernardo:** Jesús Alonso Fernandez, Natalia Lopez Martinez, Segundo Ruiz Escolar, Maria Concepción Vela Velazquez, Alejandrina Zapico Martinez. **PCHC Villaamil:** Begoña Artola Irazabal, Sheila Asenjo Martín, Maria Del Carmen Ballano Cabañas, Ana Cabot Torres, Virginia Donaire García, M. Yolanda Fernandez Martín, Pablo Gomez Alvarez, Jose Gomez Ferrnández, Rocío Guzmán Caballero, Ana Miguel Brea, Laura Muñoz Del Río, Mª Teresa Peces Paredes, Tania Ramos Geldres, Pedro Sánchez De La Calle.
